# Machine learning and complex network analysis of drug effects on neuronal microelectrode biosensor data

**DOI:** 10.1038/s41598-025-99479-7

**Published:** 2025-04-30

**Authors:** Manuel Ciba, Marc Petzold, Caroline L. Alves, Francisco A. Rodrigues, Yasuhiko Jimbo, Christiane Thielemann

**Affiliations:** 1 BioMEMS Lab, Aschaffenburg University of Applied Sciences, Aschaffenburg, Germany; 2 Institute of Mathematical and Computer Sciences (ICMC), University of São Paulo (USP), São Paulo, Brazil; 3 Department of Human and Engineered Environmental Studies, The University of Tokyo, Tokyo, Japan

**Keywords:** Data mining, Statistics, Complexity, Mammary stem cells

## Abstract

Biosensors, such as microelectrode arrays that record in vitro neuronal activity, provide powerful platforms for studying neuroactive substances. This study presents a machine learning workflow to analyze drug-induced changes in neuronal biosensor data using complex network measures from graph theory. Microelectrode array recordings of neuronal networks exposed to bicuculline, a GABA$_A$ receptor antagonist known to induce hypersynchrony, demonstrated the workflow’s ability to detect and characterize pharmacological effects. The workflow integrates network-based features with synchrony, optimizing preprocessing parameters, including spike train bin sizes, segmentation window sizes, and correlation methods. It achieved high classification accuracy (AUC up to 90%) and used Shapley Additive Explanations to interpret feature importance rankings. Significant reductions in network complexity and segregation, hallmarks of epileptiform activity induced by bicuculline, were revealed. While bicuculline’s effects are well established, this framework is designed to be broadly applicable for detecting both strong and subtle network alterations induced by neuroactive compounds. The results demonstrate the potential of this methodology for advancing biosensor applications in neuropharmacology and drug discovery.

## Introduction

Microelectrode array (MEA) biosensors with neuronal cell cultures are increasingly valuable for neurotoxicity screening^1–4^, drug testing^5–7^, physiology studies^8–10^, and disease modeling^11,12^. MEAs enable high-resolution, non-invasive monitoring of electrophysiological activity in cultured neuronal networks, providing insights into network behavior and pharmacological responses.

Analyzing MEA data involves extracting a range of features, from single-electrode measures like spike and burst rates to network-level metrics, including network burst characteristics^13^, synchrony^14,15^, and connectivity^16,17^. Connectivity analysis, in particular, enables the construction of network graphs that can be analyzed through complex network measures-key tools in neuroscience for studying interconnectivity and dynamic neuronal interactions^18–22^.

Traditional methods to assess drug effects on neuronal activity often rely on inferential statistics, which impose assumptions on data distribution, independence, and multicollinearity that may not hold in complex biological datasets^23–25^. Machine learning (ML) methods, by contrast, can uncover complex, nonlinear relationships without these constraints, making them well-suited for analyzing intricate patterns in biological data^26^.

However, ML models are often seen as “black-box” systems due to their lack of interpretability, which limits their application in fields like neuroscience and medicine where transparency is essential^27–29^. Interpretable ML techniques, such as SHapley Additive Explanations (SHAP) values, help address this issue by quantifying each feature’s contribution to predictions, allowing for biologically meaningful interpretations of ML outputs^30–32^.

SHAP values have been applied successfully in EEG and fMRI studies to interpret ML models using complex network measures^33,34^. Building upon this framework, we developed a machine-learning workflow designed to analyze spontaneous activity in in vitro neuronal networks on MEA biosensors. Our workflow systematically evaluates feature engineering parameters, including spike train bin sizes, segmentation window sizes, overlaps, and correlation methods for constructing connectivity matrices, making it adaptable to various experimental setups and reliable for pharmacological studies.

To validate this workflow, we tested it on cortical neurons grown on MEAs and treated with bicuculline (BIC), a GABA$_A$ receptor antagonist known to disrupt inhibitory neurotransmission and increase network synchrony, often resulting in an epileptiform state^15,35^. By including complex network measures as main features and synchrony as a reference feature, we assessed both the detectability of the drug effect and the biological interpretability of the ML results.

Our approach differs from previous ML applications on MEA data, such as those aiming to predict seizure-inducing actions^36^ or network development^37^. Our primary objective is to detect measurable drug effects and, crucially, to characterize these effects in biologically interpretable terms. While prior work with similar aims has used random forests (RF) for phenotype distinctions^38^ or support vector machines (SVM) for classifying drugs by their effects^39^, our workflow systematically evaluates multiple ML models, optimizes feature engineering, and incorporates a broad set of complex network measures. This comprehensive approach provides a nuanced understanding of drug effects on neuronal networks and establishes a reproducible framework for MEA biosensor applications in pharmacology. Furthermore, since our approach leverages ML to detect and interpret drug-induced effects on neuronal networks, offering advantages over traditional statistical methods by capturing complex nonlinear relationships without relying on prior assumptions, such as normality, independence, or absence of multicollinearity, ensuring robustness, scalability, and enhanced biological interpretability^40^.

Finally, we include a list of additional references in Appendix F that offers a broader context for network descriptors relevant to MEA studies and highlights potential areas for further research.

## Methods

To ensure transparency and reproducibility, the implementation of our analysis pipeline is available in our GitHub repository: https://github.com/ManuelCiba/spike-train-ml-bic.

### Electrophysiological recordings

#### Cell culture techniques

All animal procedures were reviewed and approved by the Animal Experiment Ethics Committee of the University of Tokyo (approval numbers C-12-02 and KA-14-2). The experiments were conducted in accordance with institutional guidelines for the care and use of laboratory animals at the University of Tokyo. The study also adhered to the ARRIVE guidelines for reporting animal research.

The preparation of dissociated cortical neuron cultures was based on a modified version of a previously published procedure^41^. Pregnant Wistar rats (Charles River Laboratories, Japan) were anesthetized with isoflurane and euthanized by decapitation using surgical scissors, a method approved by the Animal Experiment Ethics Committee of the University of Tokyo (approval numbers C-12-02 and KA-14-2). All procedures were conducted in accordance with institutional and national guidelines for animal welfare. At 19 days of gestation (E19), embryos were harvested and euthanized by decapitation under cold anesthesia. Cortical cells were extracted from the embryos and dissociated into individual cells using Trypsin (Life Technologies) at 37 °C for 20 min. 500,000 cells were seeded at the center of each MEA dish. Prior to seeding, the surface of the MEA dishes was coated with polyethyleneimine (PEI) (Sigma-Aldrich) overnight.

The culture medium was composed of Dulbecco’s modified Eagle’s medium (DMEM) (Life Technologies) containing 10 % heat-inactivated fetal bovine serum (FBS) (Cosmo Bio), 5 % heat-inactivated horse serum (HS) (Life Technologies), and 5-40 U/mL penicillin/streptomycin (Life Technologies). After a 30-min incubation in the MEA dishes, a 1:1 mixture of fresh culture medium and medium conditioned for three days in glial cell cultures was added. Cell cultivation was carried out in a CO$_2$ incubator at 37 °C with an atmosphere of 5 % CO$_2$ and 95 % air. Half of the culture medium was replaced every third day.

#### Microelectrode array (MEA) chip setup

**Fig. 1:**
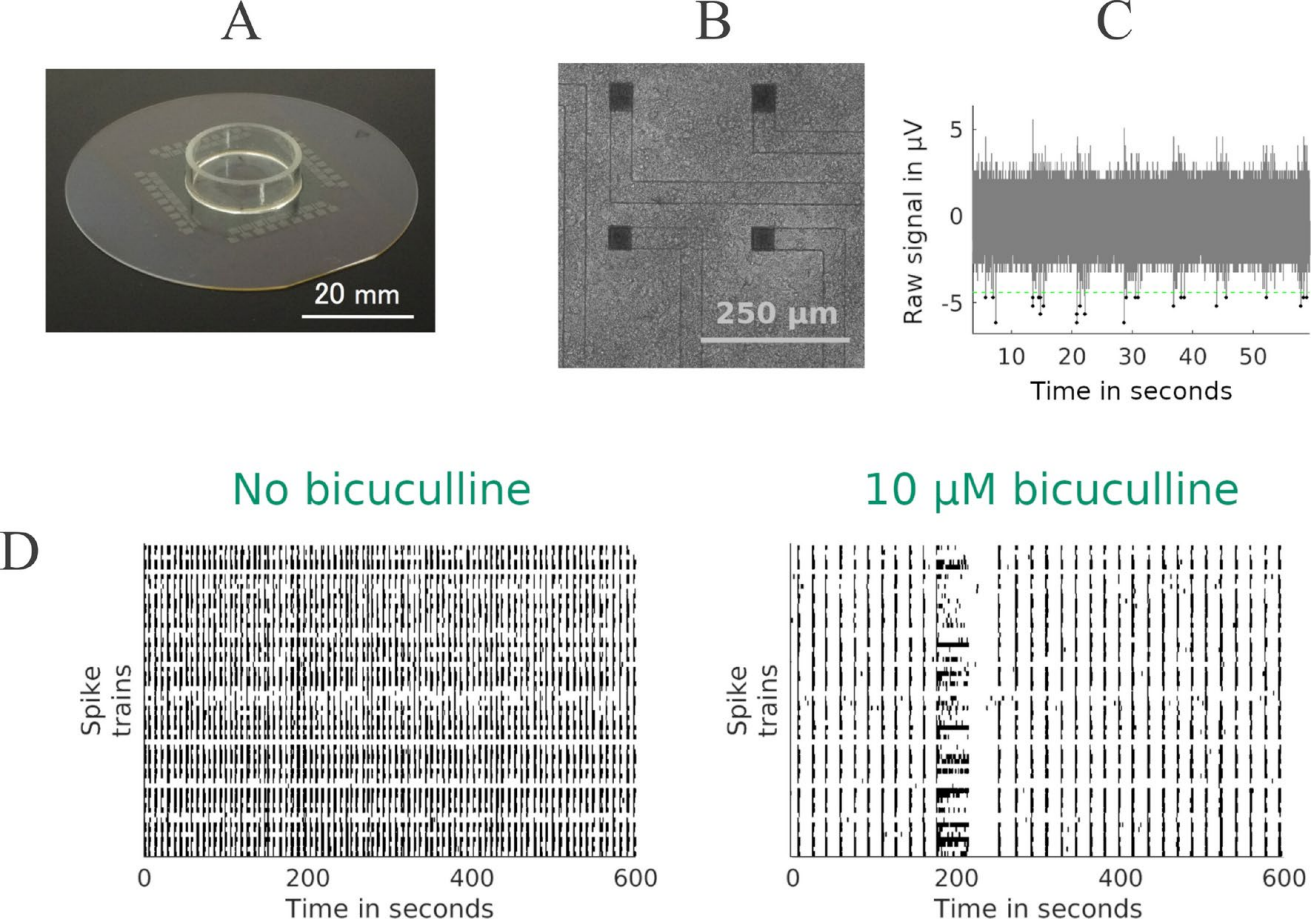
Data were acquired through electrophysiological recordings of in vitro neuronal networks cultured on MEA chips. (**A**) Depicts an MEA chip with a culture dish positioned atop the microelectrode array (image credit:^42^). (**B**) Shows a microscopic image of four out of 64 microelectrodes (visible as black squares) surrounded by cultured neurons. (**C**) Presents a 60-s segment (out of 600 s) of spontaneous activity recorded from a single electrode, along with the negative threshold employed for spike detection, a key step in the subsequent analysis. (**D**) Illustrates the spike trains from all 64 electrodes for the entire 600-s recording, both control (left) and after the application of 10 µM BIC (right).

Neuronal activity was recorded using microelectrode array (MEA) chips, enabling electrophysiological measurements of in vitro neuronal networks (Fig. 1). These recordings provided insights into neuronal connectivity and activity patterns. As shown in Fig. 1, panel (A) illustrates the MEA setup, while panel (B) highlights the placement of neurons around the electrodes. Panels (C) and (D) present representative spike train recordings from all 64 electrodes, with (C) showing a 60-s segment of spontaneous activity and (D) comparing neuronal activity before and after the application of 10 µM BIC.

#### Experimental protocol for recordings

Extracellular recordings of cortical neural networks were conducted between 21 and 54 days in vitro (div) (see Appendix A Table 2) in the MEA system (NF Corporation, Japan). Neural signals were recorded with a 25 kHz sampling frequency and band-pass filtered between 100 and 2000 Hz. All recordings were conducted in an incubator with a controlled level of 5 % CO$_2$.

Before the BIC application, spontaneous activity was recorded for 10 min following a 20-min waiting period. After the BIC application, another 20-min waiting period was followed by a 10-min recording.

It should be noted that the same cultures were also used to test an electrical stimulation protocol (“tetanic stimulation” according to^43^) before BIC application, which had already influenced spontaneous activity. However, these recordings were not included in the present study. See Appendix B for a detailed protocol description.

#### Data preprocessing and spike detection

Initially, noisy electrodes were identified and manually excluded from further analysis (see Appendix A Table 2). We then removed artifacts by setting portions of the raw signal to zero, specifically for a 6 ms interval before and a 25 ms interval after any positive peaks that exceeded a user-defined threshold unique to each MEA chip. (see Appendix A Table 2). After artifact removal, spike detection was performed by setting a negative threshold for each electrode, calculated as -5 times the standard deviation of the artifact-free signal (Fig. 1C). Finally, 18 data sets from 9 different MEA chips (9 controls, 9 with 10 µM BIC) containing spike time stamps (Figure A1 Appendix A) serve as the input data for our computational workflow. The following section describes in detail the process of further analysis and interpretation of the data.

### Machine learning workflow

We present a computational workflow to extract a set of features, incorporating complex network measures-previously established in our prior work^33,34,40,44^-to describe the structural and functional network properties. In addition to these established measures, this study introduces synchrony as a novel component, capturing the dynamics of information flow within the network, thereby expanding our methodology to provide a more comprehensive characterization of neuronal activity.

Hereafter, these features will be the base for a two-class classification process where class 0 corresponds to the native neural network and class 1 to the network disturbed by the drug BIC. Finally, an inferential statistical evaluation of possible feature modifications, as well as a machine learning (ML) classification, including the SHapley Additive exPlanations (SHAP) value method to explain the result, is performed based on our previous workflow methodology^33,34,40,44^. While our prior work introduced a novel approach by leveraging complex network measures and machine learning, it primarily focused on the Support Vector Machine (SVM) algorithm for evaluation. In this study, we expand upon that methodology by systematically testing multiple ML models, allowing for a broader classification performance and feature importance assessment.

To interpret the contribution of each feature in our model, we employed the SHapley Additive Explanations (SHAP) framework^45^, a game-theoretic approach for feature attribution in machine learning, which values quantify the effect of including or excluding a feature across different feature subsets, ensuring a fair allocation of importance. Therefore, the SHAP value $\phi _j$ for a given feature $x_j$ is defined as:

1 $$\begin{aligned} \phi _j = \sum _{S \subseteq X \setminus \{x_j\}} \frac{|S|! (|X| - |S| - 1)!}{|X|!} \left[ f(S \cup \{x_j\}) - f(S) \right] \end{aligned}$$

where:

- *S* is a subset of features excluding $x_j$.
- *f*(*S*) is the model output using only the features in *S*.
- $f(S \cup \{x_j\})$ is the model output when $x_j$ is included.
- |*S*| is the number of features in subset *S*.
- |*X*| is the total number of features.

Equation (1) quantifies each feature’s contribution by computing its marginal impact across all possible feature subsets^46,47^. The marginal impact of a feature represents the difference in model output when the feature is included versus when it is omitted. This approach ensures a fair and consistent attribution of feature importance by systematically evaluating how the inclusion or exclusion of a given feature influences the model’s output^48^. By averaging this effect over all possible subsets, SHAP ensures a fair and consistent attribution of feature importance, accounting for interactions between features and their collective influence on predictions^49^.

**Fig. 2:**
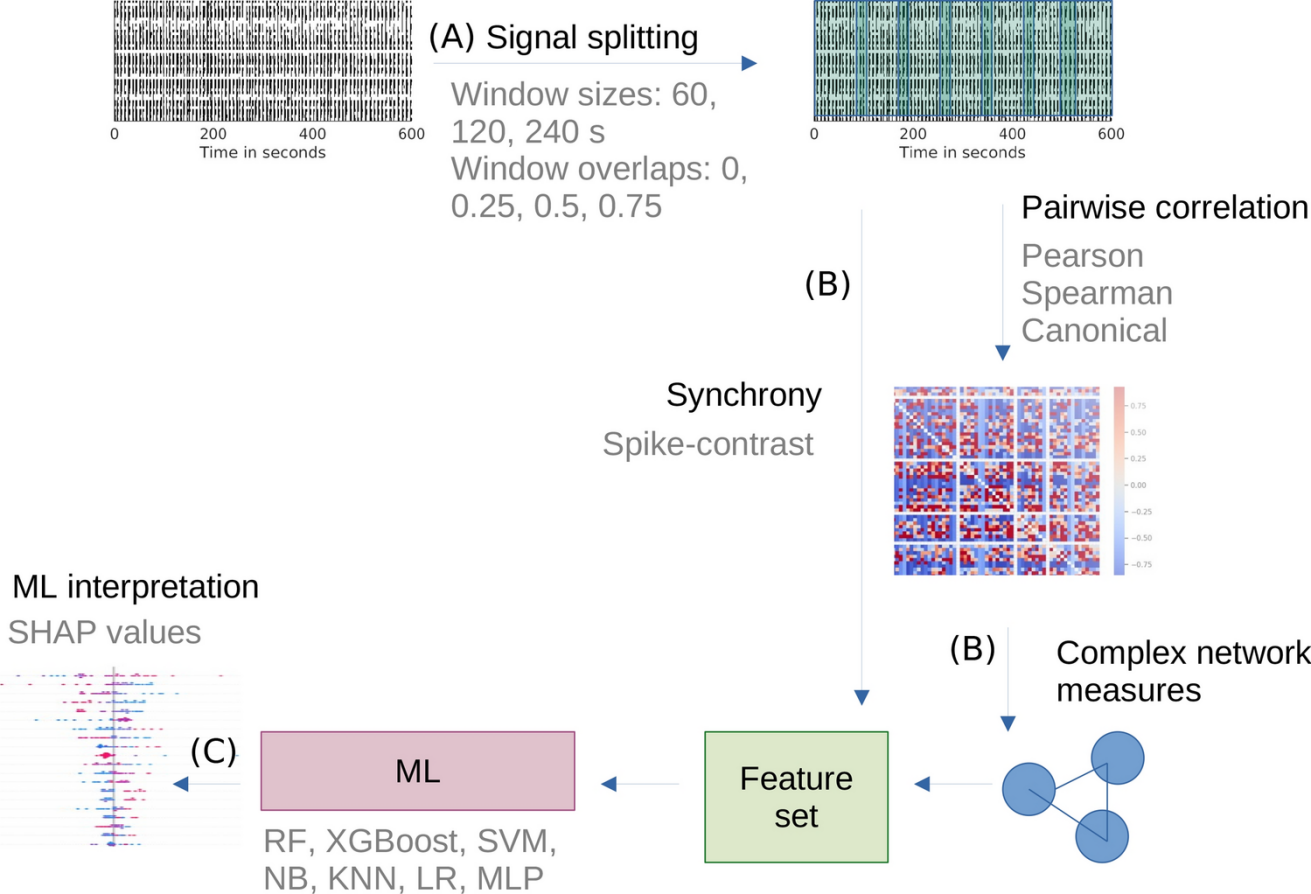
Methodology overview: (**A**) Data augmentation: the 600 s spike trains (without and with the drug) are divided into windows with varying sizes and overlaps. (**B**) Feature generation: synchrony and pairwise correlations (connectivity matrices) are computed from the segmented spike trains using various correlation methods. Complex network measures are then derived from these connectivity matrices. (**C**) Classification: different machine learning models are utilized for classification, followed by the interpretation of results.

Figure 2 illustrates the methodological workflow, divided into three parts. The first part involves the augmentation of the data set through data splitting; see Fig. 2A and “Connectivity matrices”. The second part involves feature generation. Synchrony is calculated from the spike trains, while the complex network measures are derived from the connectivity matrix., see Fig. 2B and “Connectivity matrices”, while the third part addresses the methods to reveal modifications in the network properties due to BIC, by using different ML models, see Fig. 2C and “Connectivity matrices”.

#### Data augmentation

In the context of machine learning applications, working with only 18 datasets from 9 independent networks presents a challenge due to limited data availability. To address this, we employ a sliding window approach, which segments the time series into smaller sections using either non-overlapping or overlapping windows. This technique is widely used in ML, particularly in biomedical and neuroscience research, where data scarcity is a common issue^33,50,51^.

In our study, datasets are divided into windows of 60 s, 120 s, and 240 s, with overlap levels of 0%, 25%, 50%, and 75%. Machine learning algorithms are then applied to evaluate the most effective window size and overlap configuration for classification tasks.

It is important to note that while this approach increases the number of training instances available for ML models, it does not statistically inflate the sample size due to the inherent dependency between overlapping segments. Instead, the segmentation process enhances the models’ ability to capture temporal patterns and improve generalization. To account for this dependency, we explicitly incorporate grouped leave-one-out cross-validation (LOO-CV), ensuring that all windows from the same chip remain within a single training or validation set, preventing data leakage and reinforcing model robustness.

Additionally, in our analysis, the window size determines the duration of each segment extracted from the spike train data, while window overlap controls the percentage of shared data between consecutive windows to preserve temporal continuity (e.g., a 75% overlap retains 75% of the previous window’s data). The bin size defines the temporal resolution for grouping spike counts within each window, which directly affects the connectivity matrices-these matrices quantify functional connectivity using correlation metrics. Finally, complex network measures are extracted from these matrices, providing insight into how neuronal network dynamics are modulated under bicuculline exposure.

#### Connectivity matrices

To calculate the correlation between spike trains, binary binning was applied, assigning a value of 0 for bins without spikes and 1 for bins containing at least one spike. Bin sizes of 1 ms, 10 ms, and 100 ms were used to cover different time-scales. The pairwise correlation between spike trains was computed using various metrics based on our previous workflow methodology^33,34,40,44^, including Pearson correlation, Spearman correlation, and Sparse Canonical Correlation Analysis (canonical).

Furthermore, since our methodology is based on connectivity matrices rather than raw electrode counts, it is robust to potential imbalances in the number of active electrodes. Additionally, the extracted complex network measures capture topological properties of neuronal interactions, ensuring that classification performance is driven by network features rather than the absolute number of active electrodes.

The obtained matrices were standardized to have a mean of 0 and a standard deviation of 1 to optimize their use in machine learning algorithms^33,52^. Because our experiment used paired samples (each network recorded before and after drug application), we scaled each pair together to maintain the relationship between conditions. To account for data augmentation, for each chip all windowed recordings-both pre- and post-drug application-were standardized collectively, ensuring consistency across the dataset while preserving the relationship between paired samples.

#### Feature extraction

In the next step, following the approach outlined in^40^, a complex network (or graph) was constructed from the correlation matrices by binarizing them with a threshold of 0.5. Subsequently, the following complex network measures were calculated: assortativity coefficient^53,54^, average degree of k-nearest neighbors (kNN)^55^, average shortest path length (APL)^56^, betweenness centrality (BC)^57^, closeness centrality (CC)^58^, complexity, density^59^, diameter^60^, eccentricity^61^, eigenvector centrality (EC)^62^, efficiency^63^, entropy of the degree distribution (ED)^64^, hub score^65^, k-core^66,67^, mean degree^68^, second moment of the degree distribution (SMD)^69^, and transitivity^70,71^.

Further, measures reflecting the number and structure of communities in the network were applied (described in detail in^40^). As these community measures must be transformed into a single scalar value to be included in the feature matrix, algorithms^72–74^ were applied to detect the largest community. The average path length within the largest community was calculated and a single value was received as a result. The selection of community detection metrics is based on the metrics used in^40^, which include: fastgreedy (FC)^75^, Infomap (IC)^76^, leading eigenvector (LC)^77^, label propagation (LPC)^78^, edge betweenness (EBC)^79^, spinglass (SPC)^80^, and multilevel community identification (MC)^81^. In addition, the spanning tree (SC) algorithm^82^ was used. The abbreviations were extended with the letter “A” (for average path length) to indicate the approach (AFC, AIC, ALC, ALPC, AEBC, ASPC, AMC, and ASC). The single values of the complex network measures were stored in a matrix, where each column represents a complex network measure (feature) and each row represents a MEA chip, or in our case a split window of the MEA chip. 2D matrices were created for the class with and without BIC.

A commonly used method to describe network activity and its dynamics is the measurement of synchrony. We applied the Spike-Contrast synchrony measure^15^, known for its robustness against spike detection errors compared to established measures^15^. The algorithm automatically adapts to the data, selecting the time scale that maximizes synchrony, resulting in a single synchrony value. The synchrony value was stored in an additional column of the feature matrix to combine it with the complex network measures as input for the ML-based workflow.

Finally, the feature matrices were standardized while keeping the paired sample relationship: The pre-drug features were standardized by its mean and standard deviation. The post-drug features were standardized by the mean and standard deviation of the pre-drug feature.

#### Methods to identify induced network changes

To solve the two-class problem namely the classification of the BIC and the control group, the following ML-based methods were applied based on the feature matrix containing complex network measures and the synchrony feature: SVM, k-nearest neighbors algorithm (KNN)^83^, logistic regression (LR)^84^, Random Forest (RF)^85^, Naive Bayes (NB)^86^, Multilayer Perceptron (MLP)^87^, eXtreme Gradient Boosting (XGboost)^88^ and Support Vector Machine (SVM)^89^.

The same augmented dataset was used across all machine learning algorithms. A grouped leave-one-out cross-validation (LOO-CV) approach was implemented, in which each of the 9 chips served as the validation set once, while data from the remaining chips were used for training. Specifically, the augmented recordings (pre- and post-drug) were grouped by chip, ensuring that recordings from the same chip were treated as a single unit in the validation process. For model selection and hyperparameter optimization, an internal grouped cross-validation was performed within the training set. This procedure ensures that the data from each chip is independently evaluated, preventing data leakage between the training and validation sets.

For ML performance evaluation, we used the Receiver Operating Characteristic (ROC) curve, which visualizes the relationship between true and false positive rates. The Area Under the ROC Curve (AUC) is a standard metric, with values ranging from 0.5 (random classification) to 1 (perfect classification). AUC was calculated for each of the 9 cross-validation splits, followed by bootstrapping with 1000 iterations to compute the 95% confidence interval (CI). To ensure robustness, we used the lower bound of the CI as the final AUC value for model comparison.

A possible interpretation of AUC values is as follows: an AUC of 0.5 indicates no discriminative power, meaning the model cannot distinguish between classes (e.g., with or without a condition); values from 0.7 to 0.8 are typically considered acceptable; 0.8 to 0.9 indicates excellent discrimination; and values above 0.9 are considered outstanding^90^. In this study, we interpret AUC values between 0.8 and 0.9 as indicating a significant drug effect, with values above 0.9 considered highly significant.

To identify which features are most affected by the drug, we calculated SHAP values, which systematically quantify each feature’s contribution to model predictions. By varying one feature at a time while keeping others constant, SHAP values reveal each feature’s impact, enabling the identification and prioritization of key features. This method is versatile and can be applied to any machine-learning algorithm^45^. SHAP values use cooperative game theory to increase the transparency and interpretability of ML models. They quantify the interactions between features that lead to a classification by fairly distributing the payout among the features. This method is commonly used for the interpretation of black-box results—also for the interpretation of biological and medical data sets^45,91^.

In addition to the SHAP value analysis, we applied a linear mixed model (LMM) to evaluate the distributional differences of each feature between the two classes, specifically designed to account for dependencies introduced by the sliding window technique^92–94^. Unlike traditional tests such as the Wilcoxon test, which assumes that each sample is independent, the LMM allows us to model and control for repeated measurements on the same chips across windows, enabling a more rigorous statistical analysis^95^. By including the chip as a random effect, the LMM accounts for intra-chip variability, distinguishing genuine condition effects from noise due to repeated measures.

This approach is particularly suitable for our data structure, where we have two chip conditions-BIC00 (non-BIC, control condition) and BIC10 (BIC-treated condition)-with overlapping windows within each chip that would violate the independence assumption of standard tests^96,97^. Therefore, the LMM provides a more reliable assessment of statistical significance by accounting for dependencies, yielding p-values for each complex network measure and synchrony as significance metrics. The results of these tests are detailed in “Inferential statistical evaluation of network features”, with statistical significance represented by the following annotations:

- ns: $$5.00e-02< p <= 1.00e+00$$
*: $$1.00e-02< p <= 5.00e-02$$
**: $$1.00e-03< p <= 1.00e-02$$
***: $$1.00e-04< p <= 1.00e-03$$
****: $$p <= 1.00e-04$$

## Results

### Electrophysiological recordings

Networks of primary neurons showed spontaneous activity. Recordings were only possible for a subset of electrodes (out of 64) as a neuron may not cover some electrodes or the neuron is inactive. The detected spikes from the recorded time series data are stored in 2D matrices. Each column in these matrices corresponds to an electrode on the MEA chip. The rows represent the spike times of the neurons recorded by the electrodes. If an electrode does not record any activity, the rows of a column do not contain any values, and the electrode is considered inactive. The nine chips’ active electrodes per class are shown in Appendix A Figure A1. Each time series recorded at an active electrode represents a node in the complex network calculated from the functional connectivity matrix. All chips show activity, and the number of active electrodes is similar in both classes. Therefore, we assume that the number of electrodes does not impact the classification results.

### Selection of the window size, overlapping and correlation metric

Figure A2 in Appendix C demonstrates how the choice of window size, overlap, and correlation metric impacts the performance of different machine learning models. For instance, smaller window overlaps of 0% or 25% produced optimal results for some models, such as SVM and NB. However, a 75% window overlap was chosen for further analysis, as it yielded better overall performance across most models.

The effect of bin size on performance was less consistent and can be seen better in Figure A3 in Appendix C. In this analysis, the Spike-Contrast feature was excluded because it is independent of bin size and the correlation method. The figure reveals that RF, SVM, and NB exhibited improved performance with a medium bin size of 10 ms. However, for all other models, the highest performance was achieved with the smallest bin size of 1 ms. Among the correlation metrics, Spearman and Pearson correlations outperformed the canonical correlation, with both yielding nearly identical performance levels.

**Fig. 3:**
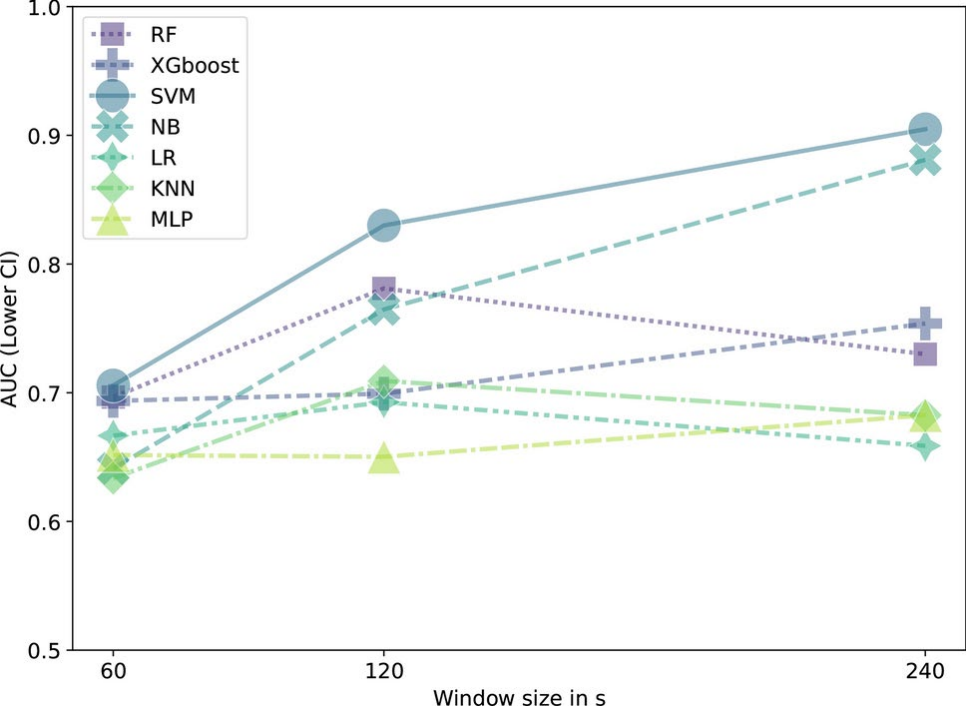
Test performance comparison of machine learning models as a function of window size. All other parameters are fixed: bin size = 1 ms, window overlap = 75 %, and correlation method = Pearson. AUC values represent the lower bound of the 95% confidence interval, calculated from the 9 cross-validation splits.

Based on these findings, we selected a 75% window overlap, a 1 ms bin size, and the Pearson correlation for further analysis, concentrating on determining the optimal window size. Figure 3 presents the test performance across various machine learning models for window sizes of 60 s, 120 s, and 240 s, using a 75% window overlap, 1 ms bin size, and Pearson correlation. A single window overlap of 75% was chosen to ensure comparability across models while optimizing overall classification performance, preventing confounding effects from varying preprocessing settings and allowing for a fair assessment of model performance under a unified framework. Results indicated that the largest window size of 240 s improved performance for most models, except for RF, LR, and KNN, which performed better with a window size of 120 s. Interestingly, when the synchrony feature Spike-contrast was excluded from the ML workflow, RF demonstrated its best performance at a window size of 240 s (see Appendix Figure A4 in Appendix C).

In terms of machine learning model performance shown in Fig. 3, SVM was the top performer, closely followed by NB, with both achieving AUC values around 0.9. RF and XGBoost performed moderately well, with AUC values above 0.7. The lowest-performing models were LR, KNN, and MLP, each with AUC values below 0.7. Notably, when the reference feature Spike-contrast was excluded, the AUC values for the best-performing models dropped from 0.9 to approximately 0.8, while RF slightly surpassed SVM and NB in performance (see attached Figure A4 in Appendix C.

In the following “SHAP values consistency”, we delve deeper into the consistency of these machine learning models by analyzing the SHAP values to understand better the feature importance and interpretability of the model’s predictions.

### SHAP values consistency

**Fig. 4:**
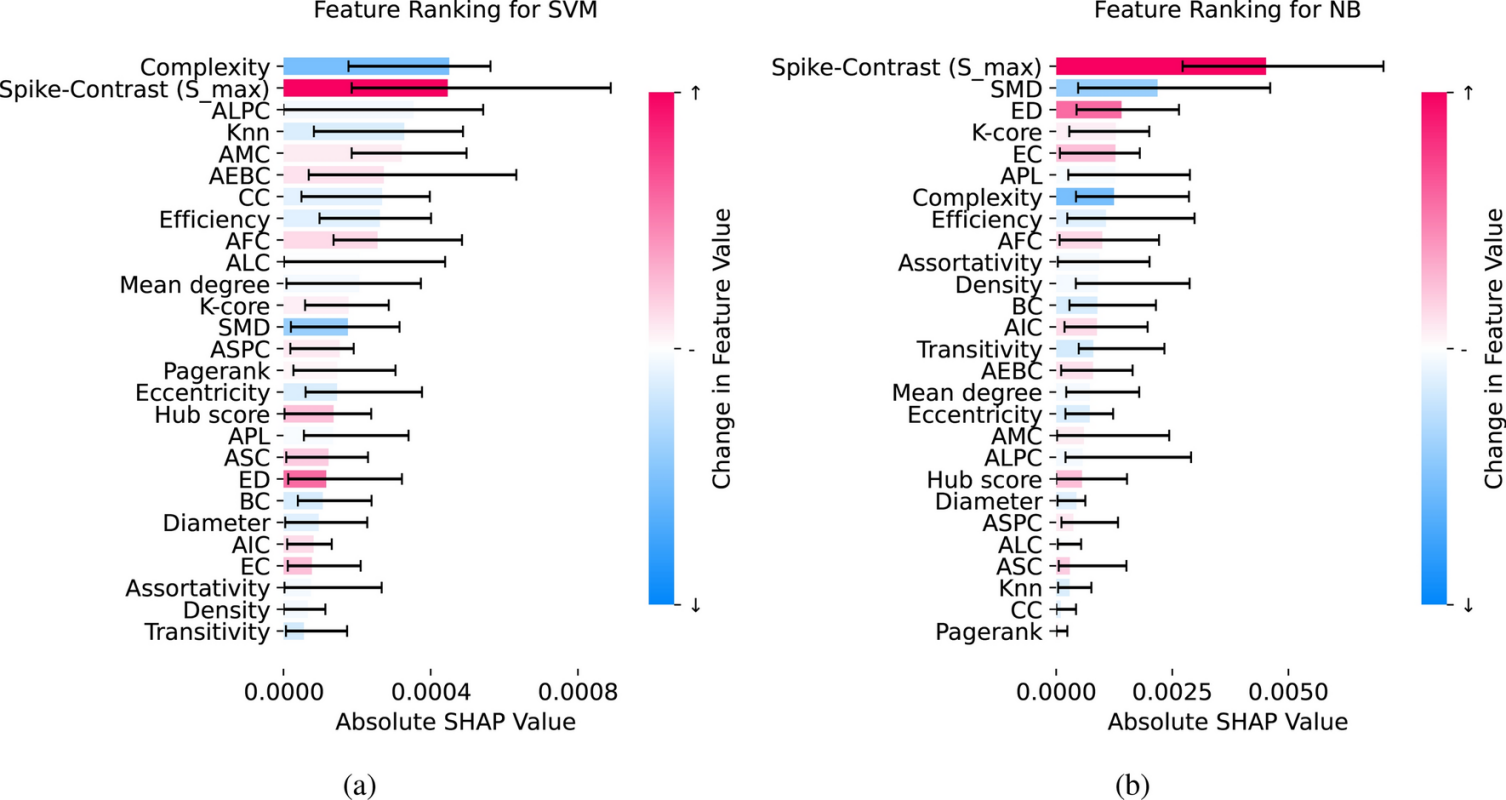
Feature importance ranking for top-performing machine learning models based on SHAP values. For each feature, the median of the absolute SHAP value for class 1 is shown, along with min and max values across 9 cross-validation splits. Color coding indicates whether features decreased or increased following BIC application.

**Fig. 5:**
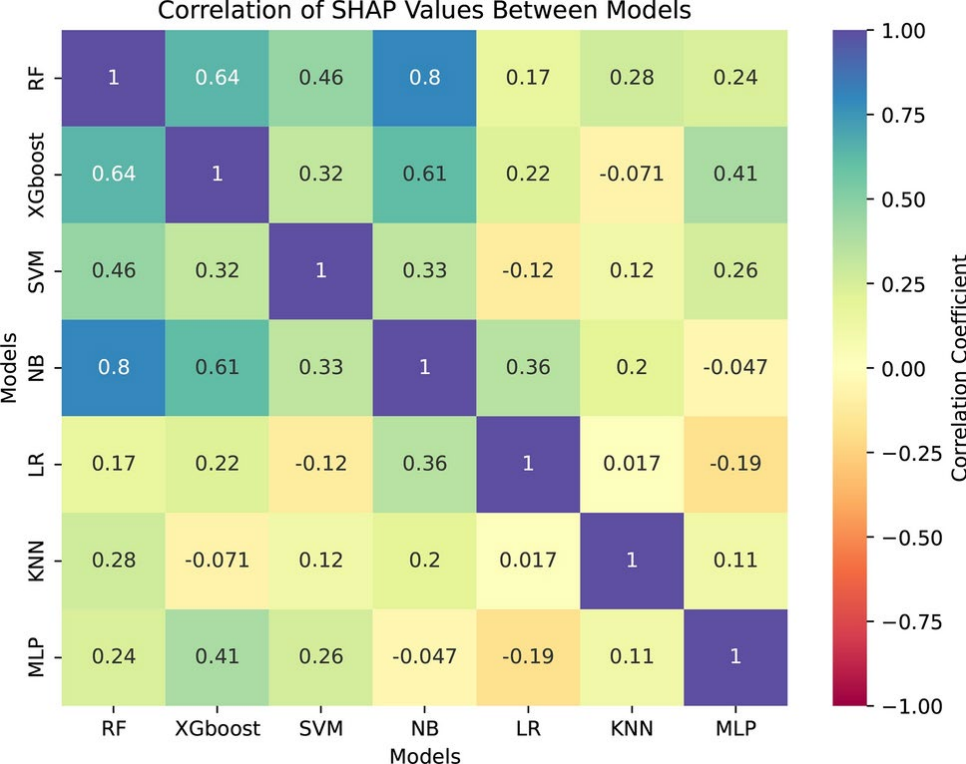
Correlation of SHAP values among different machine learning models. The heatmap displays the correlation coefficients between the median SHAP values for each model, illustrating the similarity in feature importance across models. Values closer to 1 (indicated in blue) signify high similarity in the importance assigned to features, while values closer to $-1$ (indicated in red) indicate opposing importance patterns. The color intensity reflects the strength of the correlation, providing insights into how similarly or differently the models interpret feature contributions.

We calculated the SHAP values for all machine learning models to evaluate feature importance. Figure 4 displays the feature rankings for the two best-performing models, SVM and NB. To assess the similarity of feature importance across models, we computed Pearson correlation coefficients between the feature importance vectors, as shown in Fig. 5. The highest similarity was observed between RF and NB, with a correlation of 0.8, indicating strong agreement in feature importance rankings. RF and XGBoost also exhibited notable similarity, with a correlation of 0.64. However, most pairwise comparisons between models showed lower correlations, generally below 0.5.

From the SHAP rankings presented in Fig. 4, we identified the three most influential features for each model’s predictions. These key features are summarized in Table 1. An upward arrow ($\uparrow$) indicates an increase in the feature metric following BIC application, while a downward arrow ($\downarrow$) signifies a decrease.

**Table 1:** SHAP value-based feature evaluation across machine learning models. The table highlights the top three features for each model. Arrows denote whether the corresponding feature was higher ($\uparrow$) or lower ($\downarrow$) in the BIC condition. The last column shows the p-values from the inferential statistical evaluation of the features, with four stars being the most significant and ns being the nonsignificant one. AUC values represent the lower bound of the 95% confidence interval, calculated from the 9 cross-validation splits.

AUC (lower CI)	RF	GXboost	SVM	NB	LR	KNN	MLP	P-values
	0.73	0.75	0.9	0.88	0.66	0.68	0.68	
Spike-contrast	$\uparrow$	$\uparrow$	$\uparrow$	$\uparrow$				****
Complexity	$\downarrow$	$\downarrow$	$\downarrow$					****
ASC	$\uparrow$						$\uparrow$	****
SMD				$\downarrow$				****
ED				$\uparrow$				****
Transitivity					$\downarrow$			****
APL					$\downarrow$			**
AEBC						$\uparrow$		**
BC		$\downarrow$					$\downarrow$	ns
ALPC			$\uparrow$					ns
AFC						$\uparrow$		ns
Mean degree						$\downarrow$		ns
AMC							$\downarrow$	ns
K-core					$\uparrow$			ns
AIC								***
EC								***
Hub-score								***
Knn								**
Assortativity								**
CC								**
Eccentricity								*

The synchrony metric Spike-Contrast consistently emerged as a crucial feature across the four top-performing models (RF, XGBoost, SVM, and NB). Similarly, the complexity metric ranked highly for RF, XGBoost, and SVM. Other features demonstrated less consistency in importance across the models.

Furthermore, we also assessed potential multicollinearity among the extracted complex network features by calculating the Pearson correlation matrix. This analysis allowed us to identify relationships between network measures and ensure that redundant or highly correlated features were appropriately interpreted. The correlation heatmaps for both conditions (BIC00 and BIC10) are provided in Appendix D (Figure A5), offering a comprehensive visualization of feature dependencies. While some features exhibited high correlation values, our approach mitigates potential issues arising from multicollinearity by employing machine learning models that are inherently more robust to redundant features, such as the RF algorithm.

### Inferential statistical evaluation of network features

**Fig. 6:**
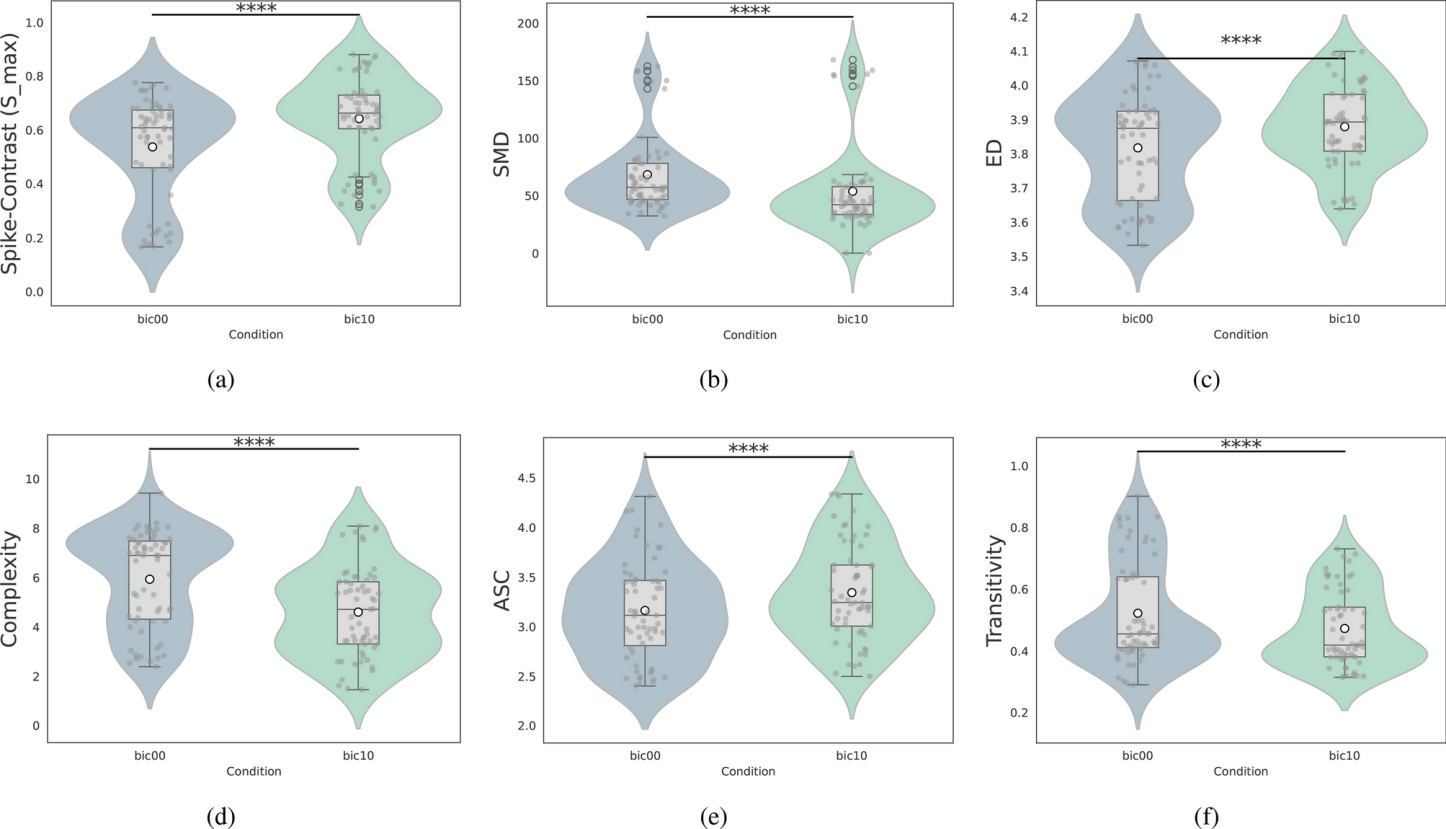
The most significant features based on LMM results across paired samples. The features with the highest significance (****) were Spike-contrast (**a**), SMD (**b**), ED (**c**), Complexity (**d**), ASC (**e**), Transitivity (**f**). The x-axis represents the two experimental conditions: BIC00 (control condition, without bicuculline) and BIC10 (bicuculline-treated condition).

Inferential statistical tests using LMM were conducted on all features in the test sampling to validate the SHAP-based feature rankings. To provide further transparency, we have included additional details on the LMM implementation in the Appendix E, describing the modeling approach and the random effect structure. Figure 6 highlights the features with the highest statistical significance (denoted by four stars), including Spike-Contrast, SMD, ED, Complexity, ASC, and Transitivity.

The results, summarized in Table 1 and Fig. 6, reveal the top three features identified by each model. Features such as Spike-Contrast, SMD, ED, Complexity, ASC, and Transitivity not only exhibited high statistical significance (four stars) but also aligned with the high SHAP values identified for the best-performing models (SVM and NB). Overall, features with higher statistical significance tended to be important for the machine learning models.

However, some inconsistencies between feature importance and statistical significance were observed. For example, SVM ranked the non-significant feature ALPC among its top three features, while several features with one to three stars (AIC, EC, Hub-score, KNN, Assortativity, CC, and Eccentricity) were not included among the top three important features for any model.

## Discussion

From our performance analysis (Appendix C), we observed that larger window sizes, when combined with the largest overlap, resulted in improved performance across most models. This can be attributed to the ability of larger windows to capture extensive oscillatory behaviors in neural firing rates, encompassing both rapid and slow fluctuations, and thus reflecting broader temporal dynamics and neural patterns^98–100^. It is well-known from studies on simulated neuronal networks that longer signal durations improve the accuracy of functional connectivity estimation^16^. Additionally, the large overlap increases data augmentation and ensures that each window contains substantial signal context, further enhancing performance for most models. Only for the best performing models (SVM and NB) smaller window overlaps would even further improve the performance, showing that the optimal pre-processing parameter choices depends on the specific model.

Furthermore, the analysis revealed that the smallest bin size of 1 ms enhanced the performance of most models. This finding aligns with previous studies, which have shown that bin sizes around 1 ms are effective in capturing biologically interpretable connectivity from spike trains^16^. Additionally, larger bin sizes, such as 100 ms, sometimes yielded network measures with interpretations that differed significantly from those obtained using 1 ms (e.g., Complexity increased due to BIC rather than decreasing).

Marginal differences of the results for different correlation metrics (Pearson and Spearman) suggest that these metrics may be less critical than the window size and overlap in capturing meaningful neural dynamics.

Overall, our selected parameters-window size of 240 s, window overlap of 75%, bin size of 1 ms, and the Pearson correlation method-can be considered a general recommendation that performed well across multiple machine learning models. However, we advise applying this workflow with varied preprocessing parameters when investigating drugs with mechanisms of action distinct from BIC, to ensure optimal parameter selection and robust results.

The comparison of SHAP values (summarized in Table 1 and ranked in Fig. 4) with inferential statistics from the LMM (Fig. 6) provided important insights into the most critical features. Spike-Contrast, our reference metric, stood out with both high SHAP values and strong statistical significance. It consistently ranked among the top three features for all models with AUC values above 0.7. This suggests that feature rankings from models with AUC values below 0.7 may not be reliable for biological interpretation.

In general, features with very high statistical significance (four stars) were also ranked high by SHAP values, indicating agreement between the two methods. However, some features ranked highly by SHAP showed no statistical significance, highlighting inconsistencies. These discrepancies suggest that while some features may be statistically significant, they do not always influence model predictions consistently across classifiers. This emphasizes the need for caution when interpreting biological significance solely based on SHAP rankings.

To interpret the biological implications, we focused on the two highest-ranked features from the best-performing models (SVM and NB, with AUC values around 0.9). These features-Spike-Contrast, Complexity, and SMD-revealed key insights into the network changes caused by BIC:

- Spike-contrast: This measure of synchrony increased significantly after BIC administration and was consistently ranked as highly important by SHAP (Table 1) and the LMM (Fig. 6). This aligns with prior studies showing that excessive synchrony, often seen in epileptic conditions, disrupts the brain’s ability to maintain functional differentiation^9,35,101^.
- Complexity: A significant reduction in Complexity was observed after BIC, supported by both SHAP and LMM results. Complexity measures the diversity of connections in the network, with higher values indicating specialized roles for certain neurons. Reduced Complexity suggests a loss of specialization and a shift toward a more uniform network structure, deviating from the small-world topology typically seen in healthy brains^102–106^. Such changes are characteristic of epileptic conditions and reflect the increased synchrony caused by BIC^107,108^.
- SMD: Lower values of SMD indicate reduced diversity in network connections, further supporting the observation of a more homogeneous and less segregated network structure.

Overall, these results are consistent with literature showing that epilepsy and BIC-induced conditions lead to less segregated, more synchronized cortical networks^109–111^. This study confirms that BIC significantly disrupts cortical network organization, resulting in hallmark patterns of epilepsy. Moreover, our machine learning-based approach successfully captured these network changes in MEA chips, demonstrating its robustness and reliability for analyzing neural dynamics.

### Limitations

One limitation of this study is the relatively small dataset, which may affect the generalizability of the findings. To address this in future work, advanced data augmentation techniques such as transfer learning can be employed, utilizing pre-trained models on similar neural datasets to enhance model performance^112^. Additionally, expanding the dataset using a multiwell MEA system could provide a more comprehensive analysis and lead to more robust conclusions. Combining these approaches with a larger sample size will facilitate more nuanced interpretations of the effects of neuroactive substances on neural network dynamics.

Another limitation lies in the experimental design, which utilized a relatively high dosage of BIC, resulting in pronounced changes in spike train patterns. While this setup effectively demonstrated the workflow’s utility, applying the methodology to data with subtler drug effects could further highlight the advantages of the ML-based workflow compared to traditional statistical methods.

Finally, when interpreting complex network measures in a biological context, it is important to acknowledge that general methods, such as Pearson and Spearman correlation, were used to estimate network connections. These techniques are not specific to neuroscience but were chosen to test the generalizability of the ML workflow. Future studies could explore alternative connectivity estimation methods more tailored to spike train data, such as Granger causality or transfer entropy, to enhance biological specificity and interpretation. For instance, the Total Spiking Probability Edges (TSPE) method^16^ has been used extensively in prior studies^11,113,114^ to capture network dynamics in spiking neural networks more effectively. TSPE not only quantifies the connection strength between pairs of spike trains but also distinguishes between excitatory and inhibitory connections. This additional information cannot be processed by the traditional complex network measures used in this work and requires alternative handling, such as constructing separate excitatory and inhibitory networks, as demonstrated in^11^. Whether incorporating these additional details into a machine learning workflow could be beneficial remains an open question for future research.

### Summary of the main findings

This study highlights the potential of ML-based approaches to analyze drug-induced effects on neuronal networks using microelectrode biosensor data. By systematically testing preprocessing parameters, scaling strategies, and feature extraction methods, we established a robust workflow tailored to paired sample designs. Our models achieved high classification performance, with AUC values reaching up to 90%, and SHAP analysis provided interpretable insights into how BIC alters network properties.

The incorporation of synchrony as a reference feature validated the plausibility of SHAP-derived feature rankings for high-performing models (AUC > 0.7), reinforcing their reliability for biological interpretation. However, we caution that some highly ranked features may lack statistical significance, highlighting the need for integrating statistical tests, such as LMM, to prioritize biologically meaningful features. This dual approach enhances confidence in the derived insights while mitigating the risks of overinterpretation.

Biologically, our findings reaffirm the role of reduced cortical network integration and segregation in epileptic states, consistent with existing literature. This underscores the disruptive impact of BIC on neural network dynamics, providing a benchmark for studying other neuroactive substances.

To ensure comparability across different ML models, we standardized preprocessing, employed AUC as a common metric, used SHAP for feature importance analysis, and validated findings with LMM. These methodological safeguards ensure that key insights remain robust despite differences in ML model performance.

Future research will extend this methodology to investigate compounds with more subtle or complex effects, exploring their influence on neuronal connectivity and dynamics. While the present study focused on a compound with well-established effects, future work will aim to demonstrate the framework’s sensitivity to more subtle pharmacological influences. By expanding its scope, this workflow could serve as a valuable tool for drug discovery, neuropharmacology, and radiation biology. Specifically, it will be applied to projects analyzing radiation-induced effects on cells using multimodal data, including microscopy images and microelectrode biosensor recordings. Additionally, future studies will examine the impact of different stimulation protocols and cell age at the time of recording on neuronal responses, further refining the interpretability of MEA-based pharmacological studies. By integrating these approaches, we aim to enhance our understanding of how diverse compounds and radiation modulate neural networks, offering deeper insights into cellular network dynamics and their responses to various perturbations.

## Supplementary Information

Supplementary Information 1.

Supplementary Information 2.

Supplementary Information 3.

Supplementary Information 4.

Supplementary Information 5.

Supplementary Information 6.

## Data Availability

Data requests can be directed to the corresponding authors and will be provided upon reasonable request, subject to institutional and ethical guidelines.
