# Supplementary material for: Machine learning and complex network analysis of drug effects on neuronal microelectrode biosensor data
Source: Sci Rep. 2025 Apr 30;15:15128. doi: 10.1038/s41598-025-99479-7 (PMC12041479; doi:10.1038/s41598-025-99479-7)
Supplement: Supplementary file 1 — Supplementary Information 1. [file 41598_2025_99479_MOESM1_ESM.pdf]

A Cell culture details

Table 2. Cell culture details for data generation and pre-processing.

Experiment name	Cell culture (Rat A/B/C, Date of seeding, dish nr.)	Cell amount	Cell age at recording	Excluded electrodes	Artefact threshold
rec20160621_no1	B, 20160525, 1	500 k	27 div	6 41	100 µV
rec20160630_no4	A, 20160510, 4	500 k	50 div	1 12 23 40 41 47 63	100 µV
rec20160701_no1	A, 20160510, 6	500 k	51 div	1 2 3 13 22 32 41 42 43 44 45 63	20 µV
rec20160702_no1	B, 20160525, 2	500 k	36 div	1 12 41 43 51 63	20 µV
rec20160702_no2	B, 20160525, 3	500 k	36 div	1 41 42 43 44 47 55 63	100 µV
rec20160704_no1	A, 20160510, 5	500 k	54 div	1 8 41 42 43 44 45 63	100 µV
rec20160803_no1	C, 20160714, 10	NA	21 div	2 6 25 29 31 32 41 45 47 54 55 63 64	100 µV
rec20160803_no2	C, 20160714, 11	NA	21 div	6 25 41 45 47 62	9999 µV
rec20160803_no3	C, 20160714, 8	NA	21 div	7 13 14 24 41 42 43 44 45 47 63	100 µV

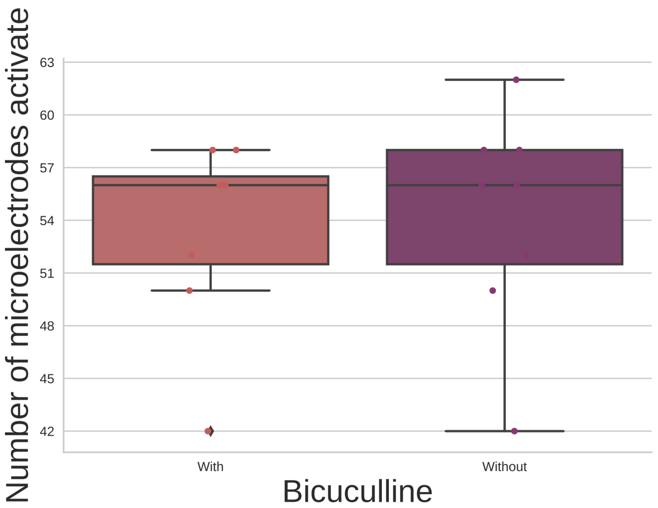

Figure A1. Number of active electrodes per chip (n=9).
