# Supplementary material for: Machine learning and complex network analysis of drug effects on neuronal microelectrode biosensor data
Source: Sci Rep. 2025 Apr 30;15:15128. doi: 10.1038/s41598-025-99479-7 (PMC12041479; doi:10.1038/s41598-025-99479-7)
Supplement: Supplementary file 2 — Supplementary Information 2. [file 41598_2025_99479_MOESM2_ESM.pdf]

B Electrical stimulation protocol

After each recording of spontaneous activity, the networks were electrically stimulated to elicit evoked activity. Additionally, tetanic stimulation was applied prior to the administration of BIC. However, in this study, only the spontaneous activity recorded before and after BIC application was analyzed (see (f) and (i) in the following paragraph).

The entire experimental protocol for each network was as follows: (a) 20 minutes resting time in the measurement system after transfer from the incubator. (b) 10-minute recording of spontaneous activity. (c) 5-minute recording of evoked activity. (e) 5-minute recording while performing tetanic stimulation. (f) 10-minute recording of spontaneous activity. (g) 5-minute recording of evoked activity. (h) Application of 10 μM BIC and waiting for 20 minutes. (i) 10-minute recording of spontaneous activity. (j) 5-minute recording of evoked activity.

Electrical stimulation during evoked activity recording was applied using pulses with a 1 V amplitude, 200 μs pulse width, and a biphasic waveform. Stimulation was administered sequentially to individual electrodes within the 8 x 8 matrix, with each electrode receiving a single pulse every second. This stimulation protocol was maintained throughout the entire 320-second recording duration.

A modified protocol inspired by[43] was used for tetanic stimulation, in which bursts of pulses were synchronously delivered across all electrodes.

Trains of 100 pulses of the same intensity (1 V) and duration (200 μs) at 100 Hz were applied 150 times at a frequency of 0.5 Hz. Note that, in contrast to the original stimulation protocol described in[43], our protocol involved a higher frequency (100 Hz vs. 20 Hz) and a greater stimulation amount (150 times vs. 10 times).
