# Supplementary material for: Machine learning and complex network analysis of drug effects on neuronal microelectrode biosensor data
Source: Sci Rep. 2025 Apr 30;15:15128. doi: 10.1038/s41598-025-99479-7 (PMC12041479; doi:10.1038/s41598-025-99479-7)
Supplement: Supplementary file 3 — Supplementary Information 3. [file 41598_2025_99479_MOESM3_ESM.pdf]

C Parameter selection

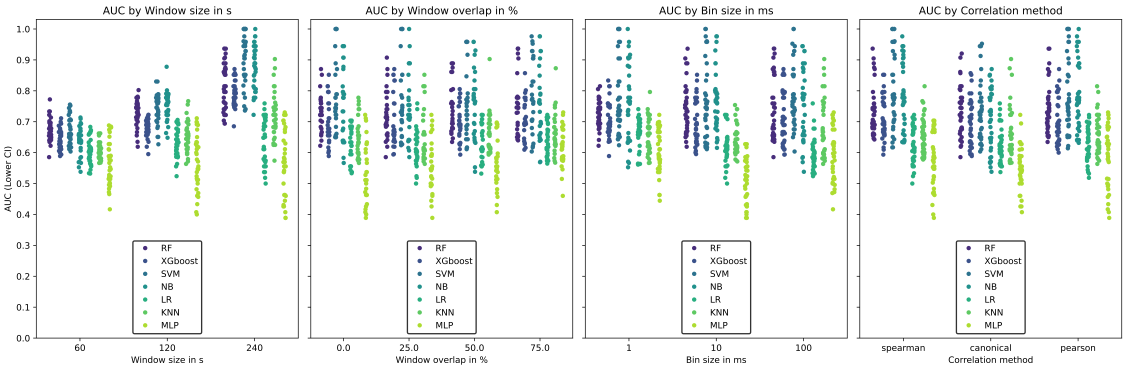

Figure A2. Comparison of test performance across machine learning models with variations in window size, overlap percentage, bin size, and correlation metric. From left to right, plots display results for window sizes, overlap percentages, bin sizes, and correlation metrics, respectively. For each fixed parameter (e.g., window size of 60 s), the AUC values for all other parameters (e.g., all bin sizes, overlaps, and correlation methods) are shown. AUC values represent the average across 9 splits.

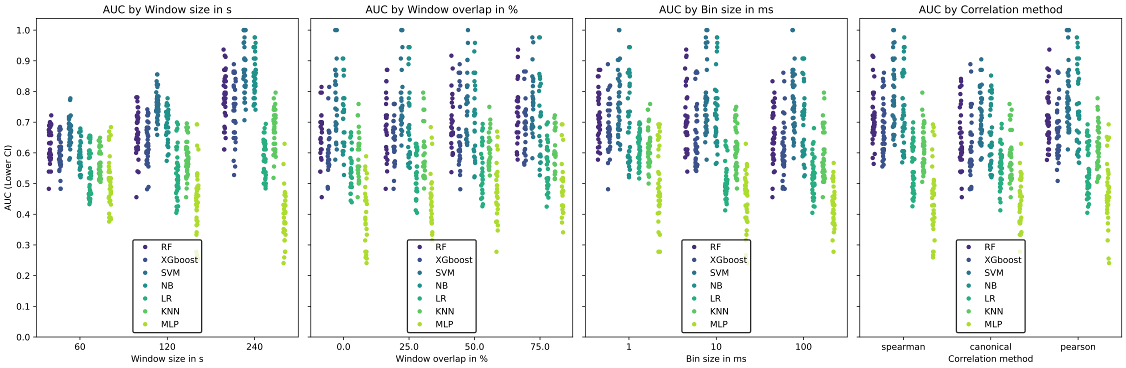

Figure A3. Comparison of test performance across machine learning models with variations in window size, overlap percentage, bin size, and correlation metric without the synchrony feature Spike-contrast. From left to right, plots display results for window sizes, overlap percentages, bin sizes, and correlation metrics, respectively. The AUC values for all other parameters (e.g., a window size of 60 s) are shown for each fixed parameter (e.g., all bin sizes, overlaps, and correlation methods). AUC values represent the average across 9 splits.

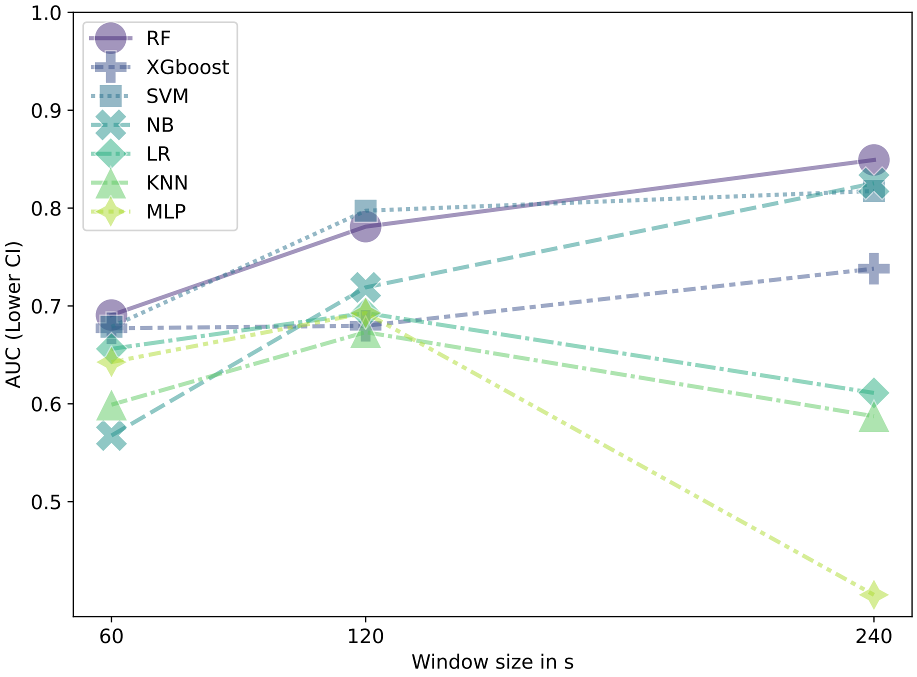

Figure A4. Test performance comparison of machine learning models without the synchrony feature Spike-contrast as a function of: bin size = 1 ms, window overlap = 75 %, and correlation method = Pearson.
