# Supplementary material for: Machine learning and complex network analysis of drug effects on neuronal microelectrode biosensor data
Source: Sci Rep. 2025 Apr 30;15:15128. doi: 10.1038/s41598-025-99479-7 (PMC12041479; doi:10.1038/s41598-025-99479-7)
Supplement: Supplementary file 4 — Supplementary Information 4. [file 41598_2025_99479_MOESM4_ESM.pdf]

D Complex network measures multicollinearity

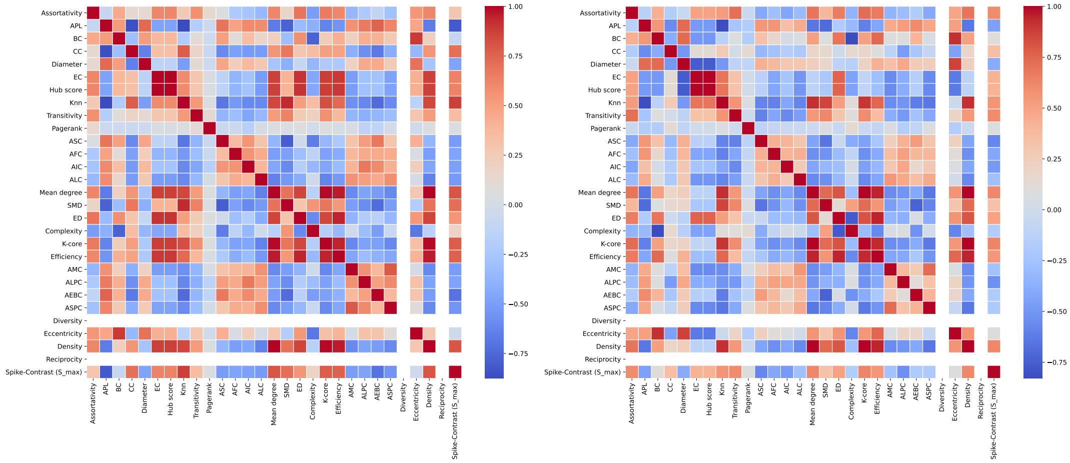

(a) (b)

Figure A5. Correlation heatmaps of extracted features for both conditions. (a) BIC00 condition and (b) BIC10 condition. The color intensity represents the strength of Pearson correlation coefficients between features.
