# Supplementary material for: Machine learning and complex network analysis of drug effects on neuronal microelectrode biosensor data
Source: Sci Rep. 2025 Apr 30;15:15128. doi: 10.1038/s41598-025-99479-7 (PMC12041479; doi:10.1038/s41598-025-99479-7)
Supplement: Supplementary file 5 — Supplementary Information 5. [file 41598_2025_99479_MOESM5_ESM.pdf]

E Linear Mixed Model (LMM) Analysis

To evaluate the differences in network measures between **pre- and post-BIC conditions**, we employed an LMM framework using the `statsmodels` package in Python[115]. Each feature was modeled as a dependent variable, with the experimental condition (BIC00 or BIC10) as a fixed effect. To account for the dependency within the data, we included the window as a random effect, ensuring that repeated measures from the same unit were properly modeled.

Each network feature was tested separately using the following model:

$$\text{Measure} \sim \text{Condition} + (1|\text{Window}) \tag{2}$$

where *Condition* represents the pre- or post-drug state, and *Window* accounts for within-chip variability. Violin plots were generated to visualize the distribution of each network feature across conditions, and statistical significance was assessed using the LMM framework.
