# Supplementary material for: Machine learning and complex network analysis of drug effects on neuronal microelectrode biosensor data
Source: Sci Rep. 2025 Apr 30;15:15128. doi: 10.1038/s41598-025-99479-7 (PMC12041479; doi:10.1038/s41598-025-99479-7)
Supplement: Supplementary file 6 — Supplementary Information 6. [file 41598_2025_99479_MOESM6_ESM.pdf]

F Additional literature

Network descriptors considered in related literature

Related Work	Data	Drug	Measurement Method	Network Activity Measures	Complex Network Measures
Ciba, et al. 2020[15]	Primary cortical neurons harvested from embryonic rats (E18 and E19)	BIC	MEA	Time-scale dependent:<br>-Cross-correlation<br>-Mutual information<br>-Spike time tiling coefficient<br>Time-scale independent:<br>-Phase synchronization<br>-Spike-Contrast<br>-A-SPIKE-synchronization<br>-A-ISI-distance<br>-A-SPIKE-distance<br>-ARI-SPIKE-distance	
Mack, et al. 2014[39]		BIC	MEA	-Firing rate<br>-Burst rate<br>-Synchrony	
Dingle, et al. 2020[116]	Primary mouse cortical neurons	Glutamatergic excitatory inputs:<br>-AP5 & NBQX<br>GABA inhibitory inputs:<br>-BIC & Picrotoxin	Genetically encoded calcium indicators & widefield microscopy image acquisition	-Ca++ frequency<br>-Global synchronization index of Ca++ transients	-Edge weight<br>-Clustering Coefficient<br>-Average path length<br>-Modularity<br>-Node degree distribution
Downes, et al. 2012[117]	Prenatal (E18) dissociated rat cortical neurons and glial cells	/	MEA	-Burst propagation time	-Number of nodes<br>-Number of links<br>-Edge density<br>-Mean path length<br>-Mean clustering coefficient<br>-Global efficiency<br>-(Conservative) Small Worldness
Cabrera-Garcia, et al. 2021[37]	Primary cultures of mouse cortical neurons	/	MEA	-Channels with spikes<br>-Network spikes<br>-Mean firing rate<br>-Interspike interval<br>-Channels with bursts<br>-Network bursts/min<br>-Mean bursting rate<br>-Burst duration<br>-Percentage of spikes in bursts<br>-Interspike interval in bursts<br>-Peak frequency in bursts<br>-Interburst interval<br>-Burst surprise<br>-STTC	-Average node degree<br>-Clustering coefficient<br>-Global efficiency

Network descriptors considered in related literature

Related Work	Data	Drug	Measurement Method	Network Activity Measures	Complex Network Measures
Srinivas, et al. 2007[118]	Hippocampal neurons from Wistar rats (postnatal)	Glutamate	MEA	-Burst frequency	-Degree<br>-Path length<br>-Clustering Coefficient
Alves, et al. 2023[33]	Autism Spectrum Disorder (ASD) patients and typical development (TD) in human brain networks	/	fMRI		-Assortativity coefficient<br>-Average shortest path length (AFC, AIC, ALC, ALPC, AEBC, ASPC, AMC)<br>-Betweenness centrality<br>-Closeness centrality<br>-Eigenvector centrality<br>-Diameter<br>-Hub score<br>-Average degree of nearest neighbors<br>-Mean degree<br>-Second moment degree distribution<br>-Entropy of the degree distribution<br>-Transitivity<br>-Complexity<br>-K-core<br>-Eccentricity<br>-Density<br>-Efficiency
Nethoff, et al. 2004[107]	Simulated hippocampus regions CA1- and CA3-like networks			-Firing rate<br>-Spike rate<br>-Refractory time<br>-Synaptic time delay<br>-Synaptic efficacy<br>-Synchrony	-Path length<br>-Portion of long distance connections<br>-Clustering Coefficient<br>-Degree distribution (heterogeneity)
